# Cerebral Blood Flow Alterations as Assessed by 3D ASL in Cognitive Impairment in Patients with Subcortical Vascular Cognitive Impairment: A Marker for Disease Severity

**DOI:** 10.3389/fnagi.2016.00211

**Published:** 2016-08-31

**Authors:** Yawen Sun, Wenwei Cao, Weina Ding, Yao Wang, Xu Han, Yan Zhou, Qun Xu, Yong Zhang, Jianrong Xu

**Affiliations:** ^1^Department of Radiology, Ren Ji Hospital, School of Medicine, Shanghai Jiao Tong UniversityShanghai, China; ^2^Department of Neurology, Ren Ji Hospital, School of Medicine, Shanghai Jiao Tong UniversityShanghai, China; ^3^GE Applied Science Laboratory, GE HealthcareShanghai, China

**Keywords:** vascular cognitive impairment, arterial spin-labeling, perfusion, MRI, cerebral blood flow, subcortical ischemic vascular disease

## Abstract

Abnormal reductions in cortical cerebral blood flow (CBF) have been identified in subcortical vascular cognitive impairment (SVCI). However, little is known about the pattern of CBF reduction in relation with the degree of cognitive impairment. CBF measured with three-dimensional (3D) Arterial Spin Labeling (ASL) perfusion magnetic resonance imaging (MRI) helps detect functional changes in subjects with SVCI. We aimed to compare CBF maps in subcortical ischemic vascular disease (SIVD) subjects with and without cognitive impairment and to detect the relationship of the regions of CBF reduction in the brain with the degree of cognitive impairment according to the *z*-score. A total of 53 subjects with SVCI and 23 matched SIVD subjects without cognitive impairment (controls), underwent a whole-brain 3D ASL MRI in the resting state. Regional CBF (rCBF) was compared voxel wise by using an analysis of variance design in a statistical parametric mapping program, with patient age and sex as covariates. Correlations were calculated between the rCBF value in the whole brain and the *z-score* in the 53 subjects with SVCI. Compared with the control subjects, SVCI group demonstrated diffuse decreased CBF in the brain. Significant positive correlations were determined in the rCBF values in the left hippocampus, left superior temporal pole gyrus, right superior frontal orbital lobe, right medial frontal orbital lobe, right middle temporal lobe, left thalamus and right insula with the *z*-scores in SVCI group. The noninvasively quantified resting CBF demonstrated altered CBF distributions in the SVCI brain. The deficit brain perfusions in the temporal and frontal lobe, hippocampus, thalamus and insula was related to the degree of cognitive impairment. Its relationship to cognition indicates the clinical relevance of this functional marker. Thus, our results provide further evidence for the mechanisms underlying the cognitive deficit in patients with SVCI.

## Introduction

Cognitive impairment after stroke is extremely common even after successful clinical recovery, a research showed the incidence of dementia after stroke is 20% at 6 months (Pendlebury and Rothwell, 2009). Vascular cognitive impairment (VCI) describes cognitive deficits that are secondary to any type of cerebrovascular disease, including both sporadic and inherited conditions, those affecting cortical and subcortical regions and those involving large and small cerebral vessels (Román et al., 2004; Rosenberg et al., 2016). Currently, small vessel disease (SVD), which includes not only lacunes in the basal ganglia and deep white matter (WM), but also more diffuse lesions in the WM, often termed leukoaraiosis (Hachinski et al., 1987), is recognized as the key mechanism of VCI; this condition is named subcortical vascular cognitive impairment (SVCI), which has an important synergy with neurodegeneration (Wardlaw et al., 2013). Except for cognitive impairment, deficits in attention, execution, verbal fluency, and in particular, set-shifting performances are common in SVDs (Roh and Lee, 2014). The other symptoms of age-related decline associated with SVDs are pseudobulbar palsy, gait disturbance, urinary incontinence and depression, which contribute to functional impairment that affect daily life task performance and result in a loss of autonomy (Banerjee et al., 2016). Thus, as the world population ages, the cognitive impairment has emerged as a growing concern in public health.

In recent years, neuroimaging is believed to enable early and accurate diagnosis and distinguish between different forms of dementia. Well-known magnetic resonance imaging (MRI) markers of SVCI include white matter hyperintensities (WMH) of presumed vascular origin, lacunes of presumed vascular origin, microbleeds and global brain atrophy (Wardlaw et al., 2013). A recent review proposed that microinfarcts, enlarged perivascular spaces and cortical superficial siderosis are imaging markers of SVCI as well (Banerjee et al., 2016). Nowadays, several imaging techniques such as diffusion and perfusion MRI, allow the investigation of blood perfusion, metabolism, activation, molecular composition and water diffusibility (Gualdi et al., 2004). Arterial spin labeling (ASL) involves the measurement of cerebral blood flow (CBF) through the direct magnetic labeling of blood water as a freely diffusable tracer (Williams et al., 1992; Detre et al., 1994). Previous studies involving the use of ASL found reductions in cortical CBF especially in frontal and parietal cortices in patients with SVCI (Schuff et al., 2009; Bangen et al., 2014). The burden of subcortical WM lesions correlated with decreases in frontal CBF and cortical atrophy (Schuff et al., 2009). Very few studies have examined the pattern of CBF reduction in relation to the degree of cognitive impairment.

In this study, we used three-dimensional (3D) ASL perfusion functional MRI in subcortical ischemic vascular disease (SIVD) subjects with and without cognitive impairment to determine the alteration of CBF distributions in subjects with SVCI and to detect the relation of the location of the decreased perfusion in the brain with the degree of cognitive impairment.

## Materials and Methods

### Participants

The current study was approved by the Research Ethics Committee of Ren Ji Hospital, School of Medicine, Shanghai Jiao Tong University, China. Written informed consent was obtained from each patient. Seventy-six participants with SIVD were recruited from patients admitted to the Neurology Department of Ren Ji Hospital from July 2012 to Jan 2015. SIVD can be defined as subcortical WM hyperintensity on T2-weighted imagings with at least one lacunar infarct, in accordance with the criteria suggested by Galluzzi et al. (2005). To be included, patients with VCI must have the following: (1) vascular cognitive impairment-no dementia (VCI-ND) diagnosed based on the Canadian Study of Health and Aging criteria (Ingles et al., 2002); and (2) vascular dementia (VaD) diagnosed on the basis of National Institute of Neurological Disorders and Stroke–Association Internationale pour la Recherche et l’Enseignement en Neurosciences (NINDS–AIREN) criteria (Román et al., 1993). Early VCI is characterized by executive function/processing speed deficits with relatively preserved memory and is less likely to produce subjective complaints, whereas Alzheimer’s disease (AD) or mixed cognitive impairment feature memory problems (Roh and Lee, 2014). Thus, we carefully excluded the participants with memory complaints.

In addition to memory complaints, the following exclusion criteria were applied: (1) cortical and/or cortico–subcortical non-lacunar territorial infarcts and watershed infarcts; (2) neurodegenerative diseases (e.g., AD and Parkinson’s disease (PD)); (3) specific causes of WM lesions (e.g., sarcoidosis, multiple sclerosis and brain irradiation); (4) cerebral hemorrhages; (5) signs of normal pressure hydrocephalus; (6) alcoholic encephalopathy; (7) education <6 years; (8) severe depression (defined by a score of ≥18 on the Hamilton Depression Rating Scale [HDRS]; Hamilton, 1960); (9) MRI safety contraindications and severe claustrophobia; (10) other psychiatric comorbidities; and (11) inability to perform neuropsychological tests. Furthermore, we have excluded participants with severe brain atrophy in the current study in order to avoid the effect of atrophy on processing fMRI data. Finally, based on cognitive status, a total of 76 recruited right-handed patients were subdivided into two groups: control group (the SIVD without cognitive impairment, *n* = 23) and the SVCI group (*n* = 53) matched on age, gender composition and years in education.

### MRI Data Acquisition

MRI was conducted using a 3.0T MRI scanner (Signa HDxt; GE HealthCare, Milwaukee, WI, USA) at Ren Ji Hospital. A standard head coil with foam padding was used to restrict head motion. Pseudocontinuous ASL (PCASL) is the continuous ASL labeling scheme that is recommended for clinical imaging (Alsop et al., 2015). PCASL perfusion images were collected using 3D fast spin-echo acquisition with background suppression and with a labeling duration of 1500 ms and post-labeling delay of 2000 ms, as suggested in a recent study (Collij et al., 2016). Repetition time (TR) = 4580 ms, Echo time (TE) = 9.8 ms, field of view (FOV) = 240 × 240 mm, matrix = 128 × 128, flip angle = 155°, slice thickness = 4 mm. CBF maps were calculated from the perfusion-weighted images using a 2-compartment model (Alsop et al., 1996) with a finite labeling duration, as described previously (Pfefferbaum et al., 2011). In addition to perfusion images, following acquisitions were also performed: (1) 3D-fast spoiled gradient recalled (SPGR) sequence images (TR = 6.1 ms, TE = 2.8 ms, TI = 450 ms, flip angle = 15°, slice thickness = 1.0 mm, gap = 0, FOV = 256 × 256 mm^2^, and slices = 166); (2) T2-fluid attenuated inversion recovery (FLAIR) sequence (TE = 150 ms, TR = 9075 ms, TI = 2250 ms, FOV = 256 × 256 mm^2^, and slices = 66); (3) axial T2-weighted fast spin-echo sequences (TR = 3013 ms, TE = 80 ms, FOV = 256 × 256 mm^2^, and slices = 34), and (4) Gradient Recalled Echo (GRE) T2*-weighted sequence (TR = 53.58 ms, TE = 23.93 ms, flip angle = 20°, matrix = 320 × 288, FOV = 240 × 240 mm^2^, slice thickness = 2 mm, NEX = 0.7, gap = 0, and slices = 72).

### Neuropsychological Assessment

Neuropsychological assessments were performed by two neurologists with sufficient experience (QX with 15 years of experience and WC with 8 years of experience) within 1 week of the MRI. During the period between the MRI and the assessment, none of the patients suffered a transient ischemic attack (TIA) or a new clinical stroke. The Montreal Cognitive Assessment (MoCA; Pendlebury et al., 2012b), Mini Mental State Examination (MMSE; Cockrell and Folstein, 1988), and a comprehensive battery of neuropsychological tests including tests of cognitive domains were designed based on a review of relevant published reports. The tests were as follows: (1) Trail-Making Tests A and B; (2) Stroop color-word test; (3) verbal fluency (category) test; (4) auditory verbal learning test (short- and long-delayed free recall); (5) Rey–Osterrieth Complex Figure Test (delayed recall); (6) Boston Naming Test (30 words); (7) Rey–Osterrieth Complex Figure Test (copy); (8) Lawton and Brody’s Activities of Daily Living (ADL) Scale Test; (9) Barthel Index (BI); (10) HDRS; and (11) neuropsychiatric inventory. To assess the cognitive status of patients, the scores obtained in each measurement of 339 normal elderly patients (Male = 140; Female = 199) in Shanghai, China were used as the normal baseline (Xu et al., 2014). They were 50–85 years of age, had >6 years of education and no serious physical and mental disease. Cognitive dysfunction was defined as −1.5 standard deviation (SD) in at least one neuropsychological test following the method described by Pendlebury et al. (2012a). The study has common clinical settings and similar neuropsychological tests with Xu et al.’s (2014) study.

For *z*-score calculation, we generated *z*-scores for the neuropsychological measures to allow a direct comparison of different tests. A *z*-score defines where a score would fall in the normal distribution of scores. It provides a simple measure by which different measures can be compared in terms of their deviation from the mean. If a *z*-score is +1.0, it represents a score 1.0 SD above the mean score. Direct comparison of performance among tests was possible for *z*-scores on all tests were based on an identical control population.

### MRI Data Assessment

Two visual rating scales for WMH severity were used in axial T2-weighted images. Two trained observers (YW and YS) independently analyzed the MRI data, who were blinded to clinical data. The degree of WMH was rated on the age-related white-matter changes (ARWMC) visual rating scale in five different regions of the bilateral hemispheres, including the frontal, temporal, parieto-occipital, basal ganglia and infratentorial areas (Wahlund et al., 2001). The total of basic ARWMC scores for each separate brain area was obtained to determine the total (t) ARWMC score. Then, it was used to measure the entire brain WMH load of each patient (Wahlund et al., 2001). T2-weighted images from all patients were also used to assess the periventricular (PV) and deep subcortical (DS) WMH separately with the Fazekas visual rating scale (Fazekas et al., 2002). Microbleeds were defined on the axial Susceptibility Weighted Imaging (SWI) sequences, as described previously (Wardlaw et al., 2013). Total brain volume was obtained from anatomical 3D T1-weighted SPGR images using statistical parametric mapping (SPM8, Wellcome Department of Imaging Neuroscience, University College, London, UK^1^) and was calculated by taking the sum of the gray and WM probabilistic tissue maps and multiplying this by the voxel volume (1 mm^3^). Intracranial volume (ICV) was calculated as the sum of total brain volume and cerebrospinal fluid (CSF).

### Analysis of MRI Data

All patients had lacunes, WMH and slight atrophy on the MRI. We performed a whole-brain voxel-based analyses (VBA) using the SPM8 program for the 3D ASL MRI data, as we described in previous research (Feng et al., 2013; Kim et al., 2013). It was implemented as follows: we co-registered the individual CBF maps to the anatomical 3D T1-weighted SPGR images. Then, the 3D T1-weighted SPGR images were segmented into probabilistic maps for gray matter (GM), WM and CSF. To account for partial inclusion of WM when GM probability was <1, CBF values were corrected according to the following: CBFcorrect = CBFuncorrect/(GM + 0.4 * WM), where CBFcorrect and CBFuncorrect represent CBF after and before partial volume correction. GM and WM are the tissue probabilities with GM + WM + CSF = 1. Based on a previous PET study, we assumed that perfusion of WM is globally 40% of that of GM perfusion (Leenders et al., 1990; Johnson et al., 2005; Du et al., 2006). We only included comparisons of CBF values in the voxels that had more than 50% of GM and WM (i.e., GM + WM >0.5) as described in a previous study (Kim et al., 2013). This should minimize the contribution of CSF and increase the statistical power (Kim et al., 2013). Later we created our own T1-template built from the averaged T1-weighted SPGR images of all the participants and spatially normalized to the standard Montreal Neurological Institute (MNI) space (a widely used spaces in the neuroscience community; Evans et al., 1993). We spatially normalized the CBFcorrect map of each subject to the standard MNI space using the same transformation parameters of T1-weighted SPGR images normalized to the T1-template, with a reslicing resolution of 2 × 2 × 2 mm^3^. Finally, the normalized CBFcorrect maps were smoothed with 6 mm full width at half-maximum (FWHM) isotropic Gaussian kernel.

Group differences between the spatially normalized and smoothed CBFcorrect (nsCBFcorrect) maps in the SVCI and control groups were tested by voxel-wise, two-sample *t*-tests with age, gender as covariates. Multiple comparison corrections were performed using the AlphaSim program in the Analysis of Functional Neuroimages software package (AFNI; NIMH, Bethesda, MD, USA^2^, Cox, 1996) as determined by Monte Carlo simulations. Statistical maps were entered into a voxel-wise two-sample *t*-test. The results were identified for thresholds at *p* < 0.01, uncorrected and a minimum cluster size of 61 voxels. Then a significant level was set at *p* < 0.05 corrected for AlphaSim. Furthermore, we correlated the nsCBFcorrect values in the whole brain with *z-scores* in each patient, to determine whether the reduction perfusion in patients with SVCI was related to the severity of cognitive impairment. Significant differences were defined as those which survived a threshold of* p* < 0.05, AlphaSim corrected (a combined threshold of *P* < 0.01 for each voxel and a cluster size >15 voxels, yielding a corrected threshold of *p* < 0.05). In addition, the value of nsCBFcorrect images could not be expressed in absolute units of milliliters per 100 g per min, because these images have been generated from the raw CBF images by several post-processing steps.

Voxel-based morphometry (VBM) analysis with diffeomorphic anatomical registration through exponentiated liealgebra (DARTEL; Ashburner, 2007) was performed using SPM8 as followed in the steps described in a previous article (Han et al., 2012). The group differences in gray matter volume (GMV) were assessed in VBN-DARTEL using two sample *t*-tests with age and gender as covariates. The significance was set at a value of *p* < 0.05 after a false discovery rate (FDR) correction.

### Statistical Analyses

All statistical analyses were performed using SPSS version 19.0 (IBM Corporation, Armonk, NY, USA). Two-sample *t*-tests were performed to assess group comparisons for the determination of intergroup demographic differences. An *χ*^2^ test was used for sex comparison. Mann–Whitney *U* test was used for comparing continuous variables if the data were not normally distributed. The significance levels were set at *p* < 0.05 for all analyses. Interobserver variability in MRI analysis by two readers for tAREMC scale score, Fazekas score and microbleeds were analyzed by calculating the interclass correlation coefficient (ICC). The values presented here correspond to the average of the analyses performed by two observers.

## Results

### Demographics, Neuropsychological Scores, and MRI Data Analysis

Table 1 shows the demographics, neuropsychological data and MRI data analyses. The groups did not differ on age, gender composition, education level and the total brain volume/ICV. The SVCI group had lower scores on the MoCA, MMSE, *z*-score than those of the controls. On the measures of tARWMC and Fazekas PV scores, SVCI group scored significantly higher than the control group. The SVCI patients also had a higher microbleeds present and microbleeds numbers (Table 1). Intraclass correlation coefficients between two readers for WMH severity and microbleeds were ICC = 0.62–0.73.

**Table 1:** **Demographic, neuropsychological data, and magnetic resonance image analysis in control and SVCI groups**.

	VCI (*n* = 53)	CON (*n* = 23)	*p* value
Gender, male/female	40/13	16/7	0.59
Age (years)	69.71 ± 9.01	67.61 ± 9.04	0.54
Education (years)	9.41 ± 4.07	11.00 ± 2.88	0.30
MMSE	25.90 ± 3.09	28.00 ± 1.23	0.005
MoCA	19.47 ± 6.41	25.05 ± 3.85	0.004
*z*-score	−1.37 ± 1.35	0.23 ± 0.56	<0.001
Total brain volume/ICV	0.63 ± 0.05	0.65 ± 0.03	0.50
tAREMC scale score	12 (7–22)	8 (1–18)	<0.001
Fazekas PV score	2.32 (1–3)	1.50 (0–3)	0.004
Fazekas DS score	2.19 (1–3)	1.75 (1–3)	0.052
Microbleeds, % present	37/53	8/23	0.004
Microbleeds, number	8 (0–57)	0 (0–22)	0.005

*SVCI, subcortical vascular cognitive impairment; CON, control group; MoCA, Montreal Cognitive Assessment, MMSE, Mini Mental State Examination; tARWMC, total Age-Related White Matter Changes, PV, periventricular; DS, deep subcortical. Note: all data were given as mean ± SD, except tARWMC and Fazekas score, i.e., data are given as median (range). Two-sample t-tests were performed to assess group comparisons. Mann–Whitney U test was used to compare continuous variables if data was not normally distributed. p-value < 0.05 was considered to be statistically significant*.

### GMV Differences Between Groups

There was no significant difference of GMV between the two groups.

### CBF Differences Between Groups

**Figure 1:**
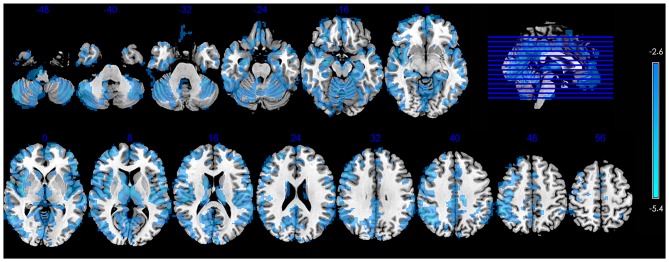
**Significant between-group differences in CBF between control subjects and those with SVCI contain the bilateral cerebral cortex (the frontal, parietal, temporal, occipital and insular lobe), periventricular (PV) gray matter (GM), basal ganglia/thalamus, brainstem and cerebellum.** The SVCI group showed decreased CBF widespread in the brain. (*p* < 0.05, AlphaSim-corrected). The *t*-score bars are shown on the right. Blue indicates SVCI < controls. Note: the left part of the figure represents the patient’s right side. SVCI, subcortical vascular cognitive impairment; CBF, cerebral blood flow.

Figure 1 shows the significant group differences in CBF. Compared with the controls, the SVCI patients had widespread hypoperfusion in the brain, including the bilateral cerebral cortex (the frontal, parietal, temporal, occipital and insular lobe), PV GM, basal ganglia/thalamus, brainstem and cerebellum. No significant hyperperfusion was found in the SVCI group.

### Correlation Between Regional CBF Values and *z* Scores in Subjects with SVCI

**Figure 2:**
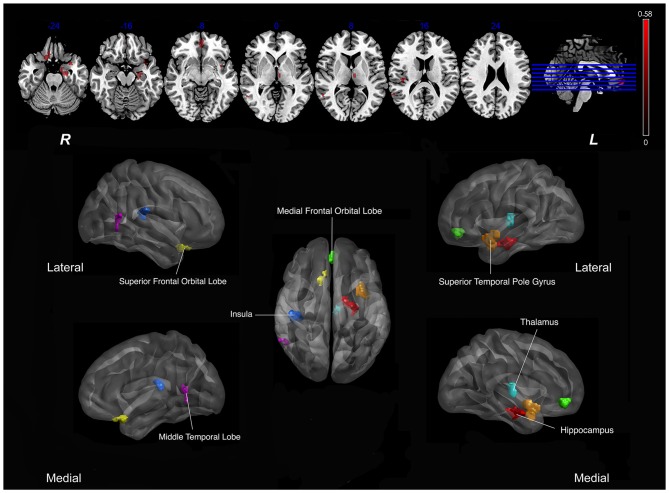
**Correlation between Regional CBF (rCBF) values and *z* scores in subjects with SVCI.** Significant positive correlations were found in the mean nsCBFcorrect values with the *z*-scores in SVCI group (*p* < 0.05, AlphaSim-corrected). Red indicates positive correlation. In the lower part of the picture, colors represent areas of the brain: red, represents the left hippocampus; orange, left superior temporal pole gyrus; yellow, right superior frontal orbital lobe; green, right medial frontal orbital lobe; dark blue, right insula; pink, right middle temporal lobe; light blue, left thalamus. Note: The left part of the figure (L) represents the participant’s left side, (R) represents the participant’s right side. SVCI, subcortical vascular cognitive impairment; CBF, cerebral blood flow.

As shown in Figure 1, SVCI group demonstrated diffuse decreased CBF in the brain, so we calculated Pearson correlations between the nsCBFcorrect values in the whole brain and *z*-scores in subjects with SVCI. Significant positive correlations were found in the nsCBFcorrect values in the left hippocampus, left superior temporal pole gyrus, right superior frontal orbital lobe, right medial frontal orbital lobe, right middle temporal lobe, left thalamus and right insula with the *z*-scores in SVCI group (Table 2 and Figure 2).

**Table 2:** **Regions showing significant correlation with *z*-scores and regional CBF in SVCI group**.

	Peak MNI coordinate region	Peak MNI coordinates			Number of cluster voxels	Peak *r* value
		*x*	*y*	*z*		
1	Left hippocampus (BA35)	−22	−14	−22	215	0.56
2	Left superior temporal pole gyrus (BA38)	−34	8	−24	97	0.53
3	Right superior frontal orbital lobe (BA11)	14	18	−24	89	0.51
4	Right medial frontal orbital lobe (BA11)	0	48	−10	129	0.48
5	Right middle temporal lobe (BA21)	54	−54	10	71	0.46
6	Left thalamus	−6	−14	10	93	0.45
7	Right insula (BA48)	34	−22	20	154	0.58

*Abbreviation: MNI, Montreal Neurological Institute; SVCI, subcortical vascular cognitive impairment; BA, Brodmann area; CBF, cerebral blood flow. (*p* < 0.05, AlphaSim-corrected)*.

## Discussion

The main aim of the current study was to investigate the pattern of CBF alteration in SVCI patients and to determine the relationship between the location of the decreased perfusion and the degree of cognitive impairment. Previous studies detected marked CBF reductions in frontal and parietal cortices in SVCI patients. Increased subcortical WM lesions were related to decreased CBF in the frontal cortex in addition to cortical atrophy (Thong et al., 2014). One study found that SVCI showed thinner cortex predominantly in the frontal cortex as well as volume reduction in the globus pallidus, putamen, and thalamus and ventricular enlargement than the control group (Thong et al., 2014). Moreover, compared to mild SVCI, moderate-serious SVCI showed thinner cortex in the parietal and lateral temporal cortices. In the WM abnormalities, the major axonal bundles including the uncinate fasciculus, internal capsule-corona radiata, and anterior section of the inferior fronto-occipital fasciculus were mainly toward to the frontal regions, which connected to the affected cortical and subcortical structures (Thong et al., 2014). It has recently been suggested that a “total SVD score”, which combines individual MRI features of SVD in one measure, may show more values than any individual marker in representation of the overall burden of tissue damage (Staals et al., 2014). Diffusion tensor imaging (DTI) studies revealed that the microstructural disruption caused by these markers may be a common end pathway that causes connectivity loss, i.e., the “disconnection syndrome” (Geschwind, 1965), thereby leading to impairments in cognitive performance (Dumas et al., 2012; Peca et al., 2013). Banerjee et al. (2016) suggested that impaired cerebral perfusion and vascular reactivity may lead to reduce neuronal function, glial and neuronal death, and volume loss. However, it is controversial in specific mechanisms leading to brain deficits, and ultimately, cognitive impairment, which remains to be explored in SIVD in the future (Jellinger, 2008). Further research should be performed to reveal the relationship between reduction perfusion and structural connectivity.

Our results were consistent with those obtained in previous studies. The areas of decreased perfusion were located in the frontal and parietal lobes, as well as the thalamus, hippocampus and insula in the deep nucleus in the brain. Some of the early symptoms in cognitive impairment in SVCI might be explained by regional reductions in CBF. For example, frontal hypoperfusion may be related to inattention and difficulties on tests of sustained attention (Fong et al., 2011). The hippocampus contributed in memory processing, as well as the amygdala and thalamus, communicates with the association cortices and has connection with the temporal lobe structures (Giap et al., 2000). The communication between multiple cerebral cortices were disrupted by abnormalities in the nuclei of these structures, leading to dysfunction in cognition and behavior and connected cortical neurons deterioration. The thalamus plays a prominent role in processing and integrating neural activity from widespread neocortical outputs and inputs (Postuma and Dagher, 2006). It is believed that the thalamus coordinates information and facilitates communication such as attention, memory and perception in several regions of the cerebral cortex (Ystad et al., 2010; Peterburs et al., 2011). At the insula, it is believed to process convergent information to produce an emotionally relevant context for sensory experience (Craig, 2009; Vilares et al., 2012).

Consistent with the previous researches, SVCI showed more serious damages in WM and more microbleeds (Werring et al., 2010). As studies report, the CT perfusion (CTP)-derived subcortical WM CBF is independently associated with WM disease severity and suggest that hypoperfusion may precede the onset of WM change; moreover, it has been reported that the primary etiology in WM disease is WM hypoperfusion rather than secondary to cortical hypometabolism (O’Sullivan et al., 2002; Huynh et al., 2008). Indeed, a recent Pulsed ASL study showed that subjects with diffuse confluent WMH had lower mean global CBF than subjects with punctiform or early stages of confluent WMH. The demonstration also helps to explain the higher risk of cognitive deterioration in elderly subjects with diffuse confluent WMH (Bastos-Leite et al., 2008). We have not detected any significant alternation perfusion in WM, which may be due to the correlation of volume effect processing, as well as the CBF in WM is much lower than in GM. It was reported that the average GM-to-WM CBF ratios were found to be 3.5 in elderly (Asllani et al., 2009). As one study proposed, WM perfusion measurements with ASL can be severely contaminated by GM perfusion signal, especially in the elderly; GM contamination is suggested to be avoidable by WM mask erosion and removal of subcortical WM voxels from the analysis (Mutsaerts et al., 2014). Further studies should be done to find the change of WM perfusion in SVCI subjects by means of PCASL.

This study had several limitations. First, the small sample size in this study might have been an issue. Second, we used clinical data instead of autopsy data for classification in participants. AD and vascular diseases are common neurological disorder in older people. Although we carefully excluded the patients who complain about memory problems, we unavoidably included people with AD-related disease. Third, in order to control the effect of GM atrophy, we used our own T1-template carefully in MRI data preprocessing, but it still might have potentially affected the results. Fourth, ASL image quality could not be ensured completely. All images were visually good. However, the labeling efficiency was not assessed formally. Furthermore, we did not perform imaging with several delay times to account for differences in travel times between groups. We used an age-adjusted delay time of 2.0 s instead of the commonly used 1.5 s, as suggested previously (Campbell and Beaulieu, 2006; Collij et al., 2016). Fifth, the CBF results might be affected by the variability of conditions of imaging, physiological state and alertness of the research participant, as well as diet and medications. Further studies are needed to clarify the effect of these factors on CBF and their contribution to between-subject variability. Finally, we have not assessed the volume of microbleeds, which might have impacted the results. Further quantitative susceptibility mapping (QSM) study will be performed to quantify the cerebral microbleeds by their magnetic susceptibility properties (Liu et al., 2012).

## Conclusion

In the current study, we used noninvasively quantified resting CBF collected using 3D ASL perfusion MRI in SVID subjects with and without cognitive impairment. Thus, we demonstrated the altered CBF distributions in the SVCI brain. The deficit brain perfusion in the temporal and frontal lobes and in the deep nucleus such as hippocampus, thalamus and insula were related to the degree of cognitive impairment in the subjects with SVCI. Its relationship to cognition indicates the clinical relevance of this functional marker. Overall, 3D ASL could become an important additional value in SVCI. Our results provide further evidence to the mechanisms underlying the cognitive deficit in patients with SVCI.

## Author Contributions

YaZ, QX and JX: conceived and designed the experiments; YS, WD and XH: performed the experiments; YS, WC and YaZ: analyzed the data; WC, YW and YoZ: contributed reagents/materials/analysis tools; YS and YaZ: wrote the article; YS: processed the figures.

## Funding

This research was supported by the National Natural Science Foundation of China (No. 81571650), Shanghai Science and Technology Committee Medical Guide Project (western medicine; No. 14411964400).

## Conflict of Interest Statement

The authors declare that the research was conducted in the absence of any commercial or financial relationships that could be construed as a potential conflict of interest.

## Footnotes

^1^http://www.fil.ion.ucl.ac.uk/spm

^2^http://afni.nimh.nih.gov/afni
